# Efeitos Anti-inflamatórios e Cardioprotetores do HDL-C: Associação com Autoanticorpos contra LDL Oxidada?

**DOI:** 10.36660/abc.20220703

**Published:** 2022-11-01

**Authors:** Estêvão Lanna Figueiredo, Ricardo Wang

**Affiliations:** 1 Instituto Orizonti Belo Horizonte MG Brasil Instituto Orizonti, Belo Horizonte, MG – Brasil

**Keywords:** Aterosclerose, HDL-Colesterol, Lipoproteínas-HDL

O acúmulo de lipoproteínas contendo apolipoproteína B (ApoB), principalmente lipoproteína de baixa densidade (LDL), na íntima arterial tem sido atribuído a um dos passos iniciais da aterogênese.^1^ Em outros estudos, a LDL oxidada e os macrófagos são os principais fatores patogênicos para o desenvolvimento da aterosclerose.^2,3^ Por sua vez, há evidências crescentes do papel protetor de autoanticorpos contra LDL oxidada (oxLDL-AboxLDL-Ab na aterogênese, fato que pode ser potencializado pela lipoproteína de alta densidade (HDL).^4,5^

Neste volume dos Arquivos Brasileiros de Cardiologia, Nunez et al.,^6^ avaliaram a hipótese de que indivíduos com níveis mais altos de HDL-C apresentariam níveis elevados de oxLDL-Ab. Trata-se de um estudo transversal que incluiu 193 indivíduos consecutivos saudáveis ≥ 18 anos e excluiu pacientes com doença arterial coronariana (DAC), acidente vascular cerebral (AVC), causas secundárias de elevação ou redução do HDL, uso de hipolipemiantes, alcoolismo e tabagismo. Os indivíduos foram submetidos a exame físico detalhado, medidas da pressão arterial e ultrassonografia de carótidas, além de análises bioquímicas de sangue periférico. Dividiu-se os participantes em tercis, de acordo com os níveis de HDL-C: baixo (< 68mg/dL: n = 59); intermediário (68-80 mg/dL: n= 71) e alto (> 80 mg/dL: n =63).

Quando comparado ao tercil mais baixo de HDL-C, o tercil mais alto apresentava mais mulheres, idades mais avançadas e maior concentração de colesterol. As atividades da lipase hepática (LH) e da proteína de transferência de éster de colesterol (CETP) estavam reduzidas; e LH e a proteína de transferência de fosfolípides (PLTP) aumentaram no tercil mais alto de HDL-C em comparação ao tercil mais baixo. Não houve diferenças significativas nos níveis da proteína C reativa de alta sensibilidade (hsCRP) e do fator de necrose tumoral (TNFα). Entretanto, os níveis de oxLDL-Ab foram significativamente maiores no grupo com HDL-C alta, em comparação ao grupo com HDL-C baixa. A análise de regressão linear revelou que os níveis de OxLDL-Ab foram influenciados pela idade, HDL-C, tercis de HDL-C, HDL-3C e ApoAI. Na análise de regressão ajustada, apenas HDL-C e ApoAI relacionaram-se independentemente aos níveis de oxLDL-Ab.^6^

A aterosclerose é uma doença inflamatória crônica da parede arterial, caracterizada pela formação de placas contendo lipídios, tecido conjuntivo e células imunológicas na íntima das artérias. A LDL oxidada seria um gatilho para ativar esta resposta imune.^7^ O estudo CANTOS comprovou, pela primeira vez, que o tratamento anti-inflamatório (no caso, com o anticorpo contra interleucina IL-1β) reduziu desfechos clínicos em pacientes com infarto agudo do miocárdio (IAM).^8^ Subsequentemente, os efeitos anti-inflamatórios da colchicina reduziram eventos em pacientes com IAM^9^ ou DAC.^10^

No estudo de Nunez et al.,^6^ os autores observaram correlação positiva e independente entre os níveis séricos da HDL-C e ox-LDL-Ab. Isto está em concordância com a hipótese de que a HDL modularia a imunidade humoral da placa aterosclerótica e mostra o papel da oxLDL-Ab como potencial marcador de doença cardiovascular. Dentre as limitações do estudo, cita-se o fato de não terem dosado as interleucinas (especialmente IL5, que induziria a liberação de ox-LDL-Ab induzida pela HDL-C). Além disso, trata-se de um estudo realizado em um único centro e com uma amostra relativamente pequena (n = 193). De toda forma, é um estudo muito elegante e que abre caminho para futuras pesquisas.

## References

[1] Quinn MT, Parthasarathy S, Fong LG, Steinberg D (1987). Oxidatively Modified Low Density Lipoproteins: A Potential Role in Recruitment and Retention of Monocyte/Macrophages During Atherogenesis. Proc Natl Acad Sci USA.

[2] Luo C, Lian X, Hong L, Zou J, Zhu Y, Huang T (2016). High uric acid activates the ROS-AMPK pathway, impairs CD68 expression and inhibits OxLDL-induced foam-cell formation in a human monocytic cell line, THP-1,”. Cell Physiol Biochem.

[3] Zhang BC, Zhang CW, Wang C, Pan DF, Xu TD, Li DY (2016). Luteolin attenuates foam cell formation and apoptosis in ox-LDL-stimulated macrophages by enhancing autophagy. Cekll Physiol Biochem.

[4] van den Berg VJ, Vroegindewey MM, Kardys I, Boersma E, Haskard D, Hartley A (2019). Anti-Oxidized LDL Antibodies and Coronary Artery Disease: A Systematic Review. Antioxidants.

[5] Skålén K, Gustafsson M, Rydberg EK, Hultén LM, Wiklund O, Innerarity TL (2002). Subendothelial Retention of Atherogenic Lipoproteins in Early Atherosclerosis. Nature.

[6] Nunez CEC, Oliveira JB, Barros-Mazon S, Zago VHS, Kaplan DB, Nakamura RT (2022). Associação positiva entre autoanticorpos contra LDL Oxidada e HDL-C:um novo mecanismo para cardioproteçao de HDL?. Arq Bras Cardiol.

[7] Engelen SE, Robinson AJB, Zurke YX, Monaco C (2022). Therapeutic strategies targeting inflammation and immunity in atherosclerosis: how to proceed?. Nat Rev Cardiol.

[8] Ridker PM, Everett BM, Thuren T, MacFadyen JG, Chang WH, Ballantyne C (2017). Antiinflammatory therapy with canakinumab for atherosclerotic disease. N Engl J Med.

[9] Tardif JC, Kouz S, Waters D, Bertrand OF, Diaz R, Maggioni AP (2019). Efficacacy and safety of low-dose colchicine after myocardial infarction. N Engl J Med.

[10] Nidorf SM, Fiolet AT, Mosterd A, Elkelboom JW, Schut A, Opstal TS Colchicine in patients with chronic coronary disease. N Engl J Med.

