# Supplementary material for: Efeitos Anti-inflamatórios e Cardioprotetores do HDL-C: Associação com Autoanticorpos contra LDL Oxidada?
Source: Arq Bras Cardiol. 2022 Nov 1;119(5):722–3. [Article in Portuguese] doi: 10.36660/abc.20220703 (PMC9750218; doi:10.36660/abc.20220703)
Supplement: Translation

# Translation: Antiinflammatory and Cardioprotective Effects of HDL-C: Association With Autoantibodies Against Oxidized LDL?

**Authors:** Estêvão Lanna Figueiredo, Ricardo Wang

**Affiliations:** 1 Belo Horizonte MG Brazil Instituto Orizonti, Belo Horizonte, MG – Brazil

**Keywords:** Atherosclerosis, Cholesterol, HDL, Lipoproteins, HDL

The accumulation of lipoproteins containing apolipoprotein B (ApoB), mainly low-density lipoprotein (LDL), in the arterial intima has been attributed to one of the initial steps of atherogenesis.^1^ In other studies, oxidized LDL and macrophages are the main pathogenic factors for the development of atherosclerosis.^2,3^ In turn, there is increasing evidence of the protective role of autoantibodies against oxidized LDL (oxLDL-AboxLDL-Ab in atherogenesis, a fact that can be potentiated by high-density lipoprotein (HDL).^4,5^

In this volume of *Arquivos Brasileiros de Cardiologia*, Nunez et al.^6^ evaluated the hypothesis that individuals with higher levels of HDL-C would have elevated levels of oxLDL-Ab. This is a cross-sectional study that included 193 consecutive healthy individuals aged ≥ 18 years and excluded patients with coronary artery disease (CAD), stroke, secondary causes of HDL elevation or reduction, use of lipid-lowering drugs, alcoholism, and smoking. The subjects underwent a detailed physical examination, blood pressure measurements and carotid ultrasound, in addition to biochemical analyzes of peripheral blood. Participants were divided into tertiles, according to HDL-C levels: low (< 68mg/dL: n = 59); intermediate (68-80 mg/dL: n=71) and high (>80 mg/dL: n=63).

Compared to the lowest HDL-C tertile, the highest tertile had more women, older ages, and higher cholesterol concentrations. Hepatic lipase (LH) and cholesterol ester transfer protein (CETP) activities were reduced, and LH and phospholipid transfer protein (PLTP) increased in the highest tertile of HDL-C compared to the lowest tertile. There were no significant differences in the levels of high-sensitivity C-reactive protein (hsCRP) and tumor necrosis factor (TNFα). However, oxLDL-Ab levels were significantly higher in the high HDL-C group compared to the low HDL-C group. Linear regression analysis revealed that OxLDL-Ab levels were influenced by age, HDL-C, HDL-C, HDL-3C and ApoAI tertiles. In the adjusted regression analysis, only HDL-C and ApoAI were independently related to oxLDL-Ab levels.^6^

Atherosclerosis is a chronic inflammatory disease of the arterial wall, characterized by the formation of plaques containing lipids, connective tissue and immune cells in the intima of the arteries. Oxidized LDL would be a trigger to activate this immune response.^7^ The CANTOS study proved, for the first time, that antiinflammatory treatment (in this case, with the antibody against interleukin IL-1β) reduced clinical outcomes in patients with acute myocardial infarction (AMI).^8^ Subsequently, the antiinflammatory effects of colchicine reduced events in patients with AMI^9^ or CAD.^10^

In the study by Nunez et al.,^6^ the authors observed a positive and independent correlation between serum levels of HDL-C and ox-LDL-Ab. This agrees with the hypothesis that HDL would modulate humoral immunity of the atherosclerotic plaque and shows the role of oxLDL-Ab as a potential marker of cardiovascular disease. One of the study’s limitations is that they did not measure interleukins (especially IL5, which would induce the HDL-C-induced release of ox-LDL-Ab). In addition, this study was carried out in a single center with a relatively small sample (n = 193). In any case, it is a very elegant study and opens the way for future research.
